# Bridging the Gap: Understanding Appalachian Patient Satisfaction in Cosmetic Rhinoplasty

**DOI:** 10.7759/cureus.62130

**Published:** 2024-06-11

**Authors:** Armein Rahimpour, Jacy Baxter, David A Denning, Peter Ray, Barry Rahman

**Affiliations:** 1 General Surgery, Marshall University Joan C. Edwards School of Medicine, Huntington, USA; 2 Plastic and Reconstructive Surgery, Marshall University Joan C. Edwards School of Medicine, Huntington, USA

**Keywords:** surgical outcomes, modified rhinoplasty outcomes evaluation, appalachian population, patient satisfaction, rhinoplasty

## Abstract

Rhinoplasty is a surgical procedure aimed at correcting both functional and aesthetic nasal deformities, addressing issues such as trauma-induced disfigurements and patient dissatisfaction with nasal appearance. Patient satisfaction is a critical outcome measure in rhinoplasty, reflecting the success of the procedure and the quality of care provided. This study investigates factors influencing patient satisfaction among Appalachian patients undergoing rhinoplasty for aesthetic reasons, considering the unique healthcare challenges faced by rural populations. A modified Rhinoplasty Outcome Evaluation questionnaire was utilized to assess patient satisfaction. Descriptive statistics and regression analyses were performed to analyze demographic characteristics, complications, re-operations, and satisfaction scores among rural and urban participants. While no significant differences were found in demographic characteristics, trends in satisfaction scores suggest potential disparities between rural and urban populations. Rural patients exhibited marginally lower satisfaction scores and higher rates of complications and re-operations, highlighting the need for targeted interventions in rural healthcare settings. Addressing geographic barriers, enhancing preoperative education and postoperative support, and fostering interdisciplinary collaboration are essential strategies to improve patient satisfaction and outcomes in rhinoplasty procedures, particularly in rural communities. Further research with larger sample sizes and qualitative methods is warranted to explore the underlying factors contributing to patient satisfaction disparities and to inform evidence-based interventions aimed at narrowing healthcare disparities and advancing health equity in rhinoplasty care.

## Introduction

Rhinoplasty is among the oldest and most intricate surgical procedures, often performed to correct both functional and aesthetic nasal deformities. The nose, as the focal point of the face, is highly susceptible to trauma, leading to disfigurements that can be distressing to patients [1]. Functional rhinoplasties may be indicated for congenital malformations, trauma, or symptomatic relief while aesthetic rhinoplasties aim to address patient dissatisfaction with nasal appearance [2]. The techniques employed in rhinoplasty are diverse, including various types of grafts, implants, osteotomies, and cartilaginous resections, and are chosen based on the surgeon's discretion and the patient’s specific needs [2].

Patient satisfaction is a critical outcome measure in rhinoplasty, given the diversity of techniques and individual patient desires. Persistent issues, such as a drooping tip or a nasal bridge hump, are common complaints that often lead to revision rhinoplasty [3]. It is essential for surgeons to understand the factors contributing to patient satisfaction to improve surgical outcomes.

Objective measures of rhinoplasty success include before-and-after photographs and pre and postoperative peak nasal inspiratory flow [4]. However, the subjective nature of patient satisfaction necessitates the use of patient-reported outcome measures (PROMs), which evaluate factors such as quality of life and satisfaction with care [5]. The Rhinoplasty Outcome Evaluation (ROE) is one such PROM, utilizing a six-question survey on a five-point scale to gauge patient satisfaction [6]. Studies have shown that factors such as previous rhinoplasty and preoperative crooked nasal bridge are linked to lower ROE scores [7]. It is important to mention that these scales are subjective and can vary among ethnic groups significantly [8].

Despite these insights, there has been limited research on aesthetic rhinoplasty satisfaction among Appalachian patients. Appalachian states are among the states with the fewest board-certified plastic surgeons [9]. This population, disproportionately affected by cardiovascular disease, cancer, and other morbidities, often faces unique healthcare challenges and exhibits distinct health behaviors [10]. Rural patients generally have decreased access to quality care, including fewer providers, increased travel requirements, and financial burdens, which can negatively impact patient satisfaction [10]. Additionally, limited access to second opinions in rural areas may further influence satisfaction with rhinoplasty outcomes [5].

This study aims to investigate the key factors influencing patient satisfaction among Appalachian patients undergoing rhinoplasty by utilizing a modified ROE.

## Materials and methods

The study was authorized by the Marshall University Institutional Review Board (IRB No. 2122668-1). In order to complete this retrospective study, patient records maintained in Cabell Huntington Hospital's electronic medical record database were reviewed. Cabell Huntington Hospital (CHH) is an American College of Surgeons-verified Level-2 Trauma Center in Huntington, West Virginia, which serves as a regional referral center for patients of the West Virginia-Kentucky-Ohio tri-state area.

The analyzed medical files belonged to patients who underwent rhinoplasty for cosmetic indication only at CHH from June 2014 to December 2022 (7.5 years). The information technology (IT) department at CHH was contacted to obtain data. The request included any patient undergoing primary rhinoplasty at CHH for aesthetic reasons. In addition, we requested patient identifiers, such as age, gender, race, MRN, date of birth, and zip code, from the IT team. The initial sample consisted of 23 patients, with current ages ranging from 18 to 55 years. All collected data were centralized using Microsoft Excel software. After collecting these parameters, the following data were extracted from the medical records: home phone number, mobile phone number, date of original operation, BMI at time of operation, tobacco use status at time of operation, and diabetes mellitus status at time of operation. Additionally, information about the primary operation was collected including complications within one year of operation, documentation of asymmetry or hematoma, presence of re-operation, type of re-operation, and indication for re-operation.

The six-question Rhinoplasty Outcome Evaluation (ROE) survey, accessed via PubMed [6], served as the basis for our study. We expanded upon this survey by adding a question, resulting in a total of seven questions aimed at a more detailed assessment of patient satisfaction (Table 1). Each question was rated on a scale from 0 to 4, with higher scores indicating greater satisfaction. The scores from the seven questions were then aggregated, with the lowest possible score being 0 and the highest possible score being 28.

**Table 1:** Modified Rhinoplasty Outcome Evaluation survey

Question	Not at all	Somewhat	Moderately	Very much	Completely
1) How well do you like the appearance of your nose	0	1	2	3	4
2) How well are you able to breathe through your nose	0	1	2	3	4
3) How much do you feel your friends and loved ones like your nose	0	1	2	3	4
4) How confident are you that your nasal appearance is the best that it can be	0	1	2	3	4
5) Would you recommend rhinoplasty for someone else?	0	1	2	3	4
Questions	Always	Usually	Sometimes	Rarely	Never
6) Do you think your current nasal appearance limits your social or professional activities	0	1	2	3	4
7) Would you like to surgically alter the appearance or function of your nose	0	1	2	3	4

Using phone numbers obtained from electronic medical records, we contacted patients and invited them to participate in the survey. We provided full disclosure to patients regarding the time required for participation and ensured anonymity. Patients were contacted no more than three times using hospital phone numbers. Sixteen patients who had undergone rhinoplasty at CHH agreed to participate in the study. During the survey, patients were verbally presented with questions from the modified ROE (Table 1), and their responses were recorded electronically. To classify the population into rural and urban categories, we utilized zip codes.

Descriptive statistics, including means, standard deviations (SDs), frequencies, and percentages, were computed to summarize participant demographics and outcome variables. Due to the small sample size and non-normal data distribution, non-parametric methods were used to examine differences between rural and urban populations. Wilcoxon rank-sum tests were utilized for continuous variables such as age, BMI, and satisfaction scores. Fisher's exact tests were applied to categorical variables, including gender, race, diabetes, tobacco use, complications, and re-operations. Exact logistic regression assessed the association between rural and urban populations and the categorical outcomes of complications and re-operations. A linear regression model analyzed satisfaction scores. Odds ratios or beta estimates with 95% confidence intervals (CIs) were calculated where appropriate. All statistical analyses were conducted using SAS version 9.4 (SAS Institute Inc., Cary, NC, USA), with statistical significance set at p < 0.05.

## Results

Descriptive statistics comparing rural and urban areas are presented in Table 2. The study included 15 participants from rural areas and 8 from urban areas. No significant differences were found between rural and urban populations in terms of age (mean age rural with ±SD= 35.1±12.0; mean age urban with ±SD= 30.8±9.6; p = 0.49) or BMI (mean rural ±SD= 29.3 ±8.2; mean urban ±SD= 27.8±7.8; p = 0.82). Satisfaction scores were marginally lower in rural participants (mean satisfaction scores in rural ±SD= 18.5± 4.9) compared to urban participants (mean satisfaction scores in urban with ±SD= 24.3±3.3; p = 0.058).

**Table 2:** Descriptive statistics comparing rural and urban areas Data presented as n (%), excluding SD. n, number; BMI, body mass index; %, percentage; SD, standard deviation

Variables	Rural (n=15)	Urban (n=8)	p-value
	Mean±SD		
Age	35.1±12.0	30.8±9.6	0.49
BMI	29.3±8.2	27.8±7.8	0.82
Satisfaction score	18.5±4.9	24.3±3.3	0.058
	Frequency (%)		
Gender			0.62
Female	12 (80.0)	5 (62.5)	
Male	3 (20.0)	3 (37.5)	
Race			0.59
White	14 (93.3)	7 (87.5)	
Black	0 (0)	1 (12.5)	
Multiple	1 (6.7)	0 (0)	
Diabetes mellitus	2 (13.33)	0 (0)	0.53
Tobacco	1 (6.7)	0 (0)	1
Complication	4 (26.7)	1 (12.5)	0.62
Re-operation	6 (40.0)	1 (12.5)	0.34

**Figure 1:**
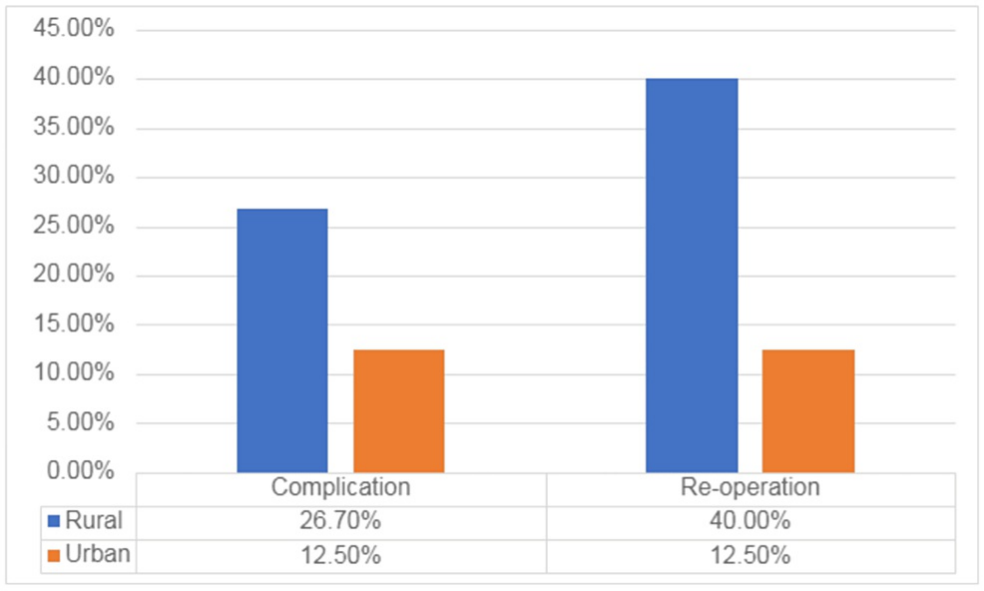
Frequencies of complications and re-operations between rural and urban populations

Regarding demographic characteristics, no significant differences were observed in gender distribution (p = 0.62), racial composition (p = 0.59), prevalence of diabetes (p = 0.53), or tobacco use (p = 1.00) between rural and urban populations. Although the incidence of complications appeared higher in the rural group (26.7%) as compared to the urban group (12.5%), and re-operations were more frequent in the rural group (40.0%) compared to the urban group (12.5%), these differences were not statistically significant (complications: p = 0.62; re-operations: p = 0.34). Figure 1 shows the complication and re-operations rate between the rural and urban populations as well.

Table 3 presents the regression model outcomes. For complications, the odds ratio was 2.45 (95% CI: 0.19, 142.9), with a p-value of 0.83, indicating no significant association between rural/urban status and complications. For re-operations, the odds ratio was 4.39 (95% CI: 0.38, 244.7), with a p-value of 0.38, suggesting no significant difference in re-operation rates between rural and urban populations. Satisfaction scores for rural participants were 5.75 points lower than for urban participants (95% CI: -11.4, 0.58), with a p-value of 0.061, indicating a borderline significant association.

**Table 3:** Comparing outcomes using logistic and linear regression models The urban population was used as the reference group.

Outcomes	Odds Ratio or Beta Estimate	95% CI	p-value
Complication	2.45	0.19, 142.9	0.83
Re-operation	4.39	0.38, 244.7	0.38
Satisfaction score	-5.75	-11.4, 0.58	0.061

## Discussion

Our study contributes to the extensive literature on patient satisfaction in rhinoplasty, with a specific focus on the Appalachian population. Rhinoplasty, as both a functional and aesthetic procedure, remains intricate and multifaceted [1,2]. Understanding patient satisfaction is crucial for optimizing outcomes and ensuring patient-centered care [3].

While our findings did not reveal significant demographic disparities between rural and urban participants, trends in satisfaction scores among rural patients are noteworthy. Rural populations face distinct healthcare challenges, including limited access to specialized care, financial constraints, and geographical barriers [4,5]. These challenges can significantly influence patient experiences and perceptions of surgical outcomes.

Although our study did not find statistically significant differences, the observed higher rates of complications and re-operations among rural participants align with existing research highlighting healthcare disparities between rural and urban areas [6]. Factors such as reduced access to follow-up care and variations in surgical techniques may contribute to these outcomes [7].

The borderline significant association between rural status and lower satisfaction scores underscores the need for targeted interventions in rural healthcare settings. Implementing patient-centered strategies, such as comprehensive preoperative education, enhanced postoperative support, and telemedicine services, can mitigate the impact of geographic barriers on patient satisfaction [8]. Furthermore, fostering interdisciplinary collaboration and involving primary care providers in the perioperative care process may enhance continuity of care and patient outcomes [11].

In addition to healthcare access, socio-cultural factors prevalent in rural communities may significantly influence patient satisfaction in rhinoplasty. Cultural perceptions of beauty, stigma surrounding cosmetic procedures, and limited health literacy could contribute to differing patient expectations and satisfaction levels [12]. To address these factors and improve patient-provider rapport, it is crucial to tailor communication strategies and deliver culturally competent care.

Preoperative counseling plays a vital role in managing patient expectations and optimizing satisfaction levels [13-14]. Research has shown that patients who receive comprehensive preoperative counseling report higher levels of satisfaction and more realistic expectations regarding the outcomes of their rhinoplasty procedures [14]. Discussions should encompass potential risks, benefits, and limitations of rhinoplasty while addressing any concerns or misconceptions. These insights are invaluable in understanding the multifaceted nature of patient satisfaction in rhinoplasty and underscore the need for tailored interventions to address individual patient needs and preferences [13-14].

While various instruments have been utilized to measure patient satisfaction and quality of life outcomes post-rhinoplasty, there remains a lack of consensus on the most appropriate tools for assessment [15]. Employing validated instruments that capture the multifaceted aspects of patient experiences, including aesthetic outcomes, functional improvements, and psychosocial impacts, is crucial [15]. By utilizing standardized assessment tools, healthcare providers can obtain more accurate and comprehensive data on patient-reported outcomes, which can inform treatment decisions and enhance patient-centered care in rhinoplasty.

Furthermore, adherence to structured perioperative care protocols has been associated with higher levels of patient satisfaction post-surgery [14]. Implementing evidence-based perioperative care protocols can improve the quality and consistency of care delivery in rhinoplasty procedures, ultimately leading to greater patient satisfaction [16].

Telemedicine services in rhinoplasty, although underexplored, have shown promise in enhancing patient satisfaction and outcomes [17]. The integration of telemedicine into the perioperative care pathway has been associated with high levels of patient satisfaction and positive outcomes [17]. These services facilitate convenient access to preoperative consultations, postoperative follow-up appointments, and patient education, particularly in rural and underserved areas where geographical barriers limit access to specialized care.

While our study focused on the Appalachian population, similar challenges in rural healthcare exist globally. Future research should explore patient satisfaction in rhinoplasty across diverse rural settings to identify common barriers and effective interventions. Large-scale prospective studies with longitudinal follow-up are warranted to validate our findings and inform evidence-based practices.

Limitation

One limitation of our study is the relatively small sample size, which may limit the generalizability of our findings. Additionally, the use of a modified Rhinoplasty Outcome Evaluation questionnaire, while informative, may not capture all dimensions of patient satisfaction comprehensively. Finally, as this is a survey-based study, usually, responders are not a good set of the population of interest and are usually the extremes.

Further study

Further investigation into the specific factors contributing to the observed differences in satisfaction scores between rural and urban populations is warranted. Longitudinal studies with larger sample sizes could explore the impact of preoperative counseling, perioperative care protocols, and postoperative follow-up strategies on patient satisfaction outcomes. Qualitative research methods, such as interviews or focus groups, may provide deeper insights into the socio-cultural factors influencing patient expectations and experiences in rhinoplasty, particularly in rural communities. Moreover, comparative studies across different geographic regions and healthcare systems can elucidate universal and context-specific determinants of patient satisfaction in rhinoplasty procedures.

## Conclusions

In conclusion, our study underscores the nuanced relationship between rural residency and patient satisfaction in rhinoplasty. Despite comparable demographic characteristics, rural patients exhibited trends of lower satisfaction scores and higher rates of complications and re-operations. Addressing the multifaceted challenges faced by rural populations is crucial for optimizing patient outcomes and narrowing healthcare disparities. By adopting patient-centered approaches and fostering interdisciplinary collaboration, healthcare providers can enhance the quality of care and improve patient satisfaction in rhinoplasty procedures, ultimately advancing health equity.
